# Systemic Lupus Erythematosus Patients Contain Significantly Less IgM against Mono-Methylated Lysine than Healthy Subjects

**DOI:** 10.1371/journal.pone.0068520

**Published:** 2013-07-16

**Authors:** Sha Guo, Ying Liu, Younan Ma, Qing Zhao, Liping Zhu, Yuehu Shao, Fengying Gao, Fengqi Wu, Ruitong Gao, Wei Zhang

**Affiliations:** 1 Department of Immunology, Institute of Basic Medical Sciences, Chinese Academy of Medical Sciences, School of Basic Medicine, Peking Union Medical College, Beijing, People’s Republic of China; 2 Department of Research and Development, Absea Biotechnology Ltd, Beijing, People’s Republic of China; 3 Department of Internal Medicine, Pediatric Hospital of Sanhe, Langfang City, Hebei Province, People’s Republic of China; 4 Department of Pediatric Rheumatology, Capital Institute of Pediatrics, Beijing, People’s Republic of China; 5 Department of Nephrology, Peking Union Medical College Hospital, Chinese Academy of Medical Sciences, Peking Union Medical College, Beijing, People’s Republic of China; Cordelier Research Center, INSERMU872-Team16, France

## Abstract

Post-translational modifications on proteins are important in biological processes but may create neo-epitopes that induce autoimmune responses. In this study, we measured the serum IgG and IgM response to a set of non-modified or acetyl- and methyl-modified peptides corresponding to residues 1–19 of the histone 3 N-terminal tail in systemic lupus erythematosus (SLE) patients and healthy subjects. Our results indicated that the SLE patients and healthy subjects produced antibodies (Abs) to the peptides, but the two groups had different Ab isotype and epitope preferences. Abs to the non-modified form, H3_1–19_, were of the IgG isotype and produced by SLE patients. They could not recognize the scrambled H3_1–19_, which contained the same amino acid composition but a different sequence as H3_1–19_. In comparison, healthy subjects in general did not produce IgG against H3_1–19_. However, about 70% of the healthy subjects produced IgM Abs against mono-methylated K9 of H3_1–19_ (H3_1–19_K9me). Our further studies revealed that ε-amine mono-methylated lysine could completely inhibit the IgM binding to H3_1–19_K9me, but lysine had no inhibitory effect. In addition, the IgM Abs could bind peptides containing a mono-methylated lysine residue but with totally different sequences. Thus, mono-methylated lysine was the sole epitope for the IgM. Interestingly, SLE patients had much lower levels of this type of IgM. There was no obvious correlation between the IgM levels and disease activity and the decreased IgM was unlikely caused by medical treatments.We also found that the IgM Abs were not polyreactive to dsDNA, ssDNA, lipopolysaccharide (LPS) or insulin and they did not exist in umbilical cord serum, implying that they were not natural Abs. The IgM Abs against mono-methylated lysine are present in healthy subjects but are significantly lower in SLE patients, suggesting a distinct origin of production and special physiological functions.

## Introduction

Systemic lupus erythematosus (SLE) is an autoimmune disease characterized by the production of various autoantibodies (autoAbs). Many of these antibodies (Abs) are reactive to components in the cell nuclei, such as dsDNA, nucleosomes, Sm, La, Ro, etc. [1], [2]. As the major components of nucleosomes, histones are also common autoantigens in SLE. These autoAbs form immune complexes with the antigens, which leads to excessive activation of complement and phagocytes, resulting in severe inflammation and multiorgan damage.

Histones can be grouped into linker histones (H1), core histones (H2A, H2B, H3 and H4) and other variants. The core histones are small basic proteins consisting of a globular domain and a flexible N-terminal tail, which comprises about 25–30% of the mass of the individual histones. Two copies of each core histone are assembled into an octamer that has 146 base pairs of DNA wrapped around to form the nucleosome core. The histone N-terminal tails protrude from the nucleosome and are exposed on the surface [3]–[5]. So far, autoAbs to all five of the histones have been found in SLE patients and lupus mouse models [2], [6]. Most of these autoAbs recognize epitopes exposed on nucleosome surfaces, such as the 22–42 residues of H1′, 1–25 residues of H2B, 1–21 residues of H3, and 1–29 residues of H4. There are fewer autoAbs against epitopes that are not located on histone tails, such as residues 65–85 of H2A and residues 40–55 of H3 [7]–[11].

Histones can be extensively modified by acetylation, methylation, phosphorylation, ubiquitination, sumoylation, ADP-ribosylation, deimination and proline isomerization [12]–[16]. Most of the post-translational modifications are found on the histone N-terminal tails. These modifications influence the overall structure of chromatin and play fundamental roles in many biological processes that are involved in the manipulation and expression of DNA, including cell development, differentiation, proliferation and apoptosis. Post-translational modifications on histones or other molecules may also create neo-self epitopes that can be recognized by antigen receptors on T cells or B cells and contribute to the development of autoimmunity in genetically predisposed individuals [17], [18]. Plaué *et al.* reported that ubiquitinated H2A and H2B were targets for SLE autoAbs [19]. van der Vlag *et al.* reported that apoptosis-associated histone modifications generated the epitopes H2BK12ac, H3K27me3, H4K8ac3, H4K12ac3 and H4K16ac3 that were recognized by autoAbs in SLE patients and lupus mice [20]–[22].

There are many sites on the 1–19 residues of the H3 N-terminal tail that can be modified to generate neo-epitopes. Although autoAbs to the non-modified H3 N-terminal tail have been reported [8], the existence of autoAbs to the modified H3 1–19 N-terminal tail is unknown. One study reported H3K9me3S10ph in an isolated case from a patient who had discoid lupus erythematosus and chronic lymphocytic leukemia [23].

In this study, we tested the reactivity of IgG and IgM from SLE patients and healthy subjects against a set of synthetic peptides corresponding to the 1–19 residues of the H3 N-terminal tail with or without methylation and acetylation. We found that both SLE patients and healthy controls could produce Abs to the peptides, but the two groups had different isotype and epitope preferences. IgG Abs were mainly produced by SLE patients with specificities to the non-modified peptide, whereas the healthy controls contained mostly IgM Abs recognizing epitopes containing mono-methylated lysine. Interestingly, SLE patients had significantly lower levels of this type of IgM than the healthy subjects.

## Materials and Methods

### Ethics Statement

This study was performed in accordance with the Declaration of Helsinki and approved by the ethic committees of Institute of Basic Medical Sciences, Chinese Academy of Medical Sciences (Approval ID 012–2012, Institutional Review Board of IBMS, CAMS). All the personal privacy was well protected throughout the work. All the serum samples used in this work were leftover samples after clinical examinations of patients or routine health check of healthy people. As these samples were treated as abandoned samples and used in anonymous codes, the informed consent was exempted. Patient’s clinical information was obtained through a doctor who had the master key of coding and he would strictly abide by the confidentiality agreement. The umbilical cord blood samples were treated as medical waste and could be disposed by the hospitals. They were also used anonymously and coded without identifiable information.

### Patients and Healthy Controls

62 pediatric-onset SLE (pSLE) patients (11 males and 51 females; mean age 10.69±3.12 ranging from 2 to 14 yrs old) were recruited between 2001 and 2012 from the Department of Pediatric Rheumatology, Capital Institute of Pediatrics, Beijing, China. 75 adult SLE (aSLE) patients (11 males and 64 females; mean age 35.00±11.19 ranging from 19 to 67 yrs old) were recruited in 2012 from the Department of Nephrology, Peking Union Medical College Hospital, Beijing, China. Information for the patients and healthy controls is listed in the Table S1. All of the patients met at least 4 classification criteria from the American College of Rheumatology [24]. Patients who were undergoing treatments when their blood was collected were listed in the Table S2. 36 juvenile idiopathic arthritis (JIA) patients (8 males and 28 females; mean age 10.86±2.75 yrs old) and 26 patients with other rheumatoid diseases (RD), including juvenile ankylosing spondylitis (2 males and 2 females; mean age 9.50±2.08 yrs old), Henoch-Schonlein purpura (2 males and 9 females; mean age 8.36±3.01 yrs old), idiopathic thrombocytopenic purpura (1 female; 9 yrs old), Kawasaki disease (1 female; 1 yr old), mixed connective tissue disease (1 female; 10 yrs old), juvenile dermatomyositis (1 male and 5 females; mean age 11.83±1.47 yrs old) and Behcet’s disease (2 females; mean age 11.50±0.71 yrs old) were recruited between 2001 and 2012 from the Department of Pediatric Rheumatology, Capital Institute of Pediatrics. The healthy controls were people who had undergone routine health checkups. None of the controls had any rheumatologic conditions when recruited. Umbilical cord blood was collected from newborns in the Maternity Hospital of Sanhe, Langfang City, Hebei Province, China.

Sera from SLE patients and healthy controls were obtained from whole blood and stored at −80°C until use.

### Peptides and Histones

The peptides (Table S3) were synthesized by Scilight-peptide Inc. (Beijing, China). All of the peptides had a cysteine residue at the end. The peptides were further cross-linked onto BSA through their cysteine residue (Absea Biotechnology Ltd., Beijing, China). The sodium dodecyl sulfate-polyacrylamide gel electrophoresis (SDS-PAGE) gel of the BSA cross-linked peptides is shown in Figure S1. Coupling H3_1–19_ and H3_1–19_K9me peptides onto Sepharose CL-4B beads was performed by Absea. The beads were finally suspended in PBS/0.1% NaN_3_ as a 50% slurry. The histones were obtained by acid extraction from Hela cells as described [25].

### Detection of Anti-peptide Abs by ELISA

96-well microtiter plates (Kelongda Institute, Beijing, China) were coated with H3 peptides cross-linked to BSA (1 µg/ml according to the BSA concentration, 100 µl/well) at 4°C overnight in coating buffer containing 15 mM Na_2_CO_3_, 35 mM NaHCO_3_, pH 9.6. After washing with PBS containing 0.05% Tween-20 (PBST), the plates were blocked with 200 µl of PBST containing 2% BSA for 2 h at room temperature. Then, 100 µl/well of the serum samples (1∶100 diluted in PBST containing 2% BSA) were added and incubated at room temperature for 1 h. After washing, the mouse anti-human IgG monoclonal Ab (mAb) KT47 or mouse anti-human IgM mAb KT16 (Absea) diluted at 1∶1000 was added. After incubating at room temperature for 1 h, the plates were washed with PBST and HRP-conjugated goat anti-mouse IgG (Product No. A2554. Sigma-Aldrich Co, St. Louis, MO, USA) diluted at 1∶5000 was added. After incubating at room temperature for 1 h, the wells were washed and the substrate 2, 2′-azino-bis-3-ethylbenzothiazoline-6-sulphonic acid (ABTS, Product No. 0400. Amresco, Solon, OH, USA) was added. Color was developed for 20 min (for IgM) or 40 min (for IgG), and the OD values were read at 405nm.

### Measurement of Serum IgM Concentrations by Sandwich ELISA

96-well microtiter plates (Kelongda) were coated with 100 µl/well of KT16 as a capture Ab (2.5 µg/ml) at 4°C overnight. The plates were then washed with PBST and blocked with 200 µl/well of PBST containing 2% BSA for 2 h at room temperature. The samples diluted in PBST containing 2% BSA (1∶1600 for adult serum IgM; 1∶800 for pediatric serum IgM) were then added. After incubating at room temperature for 1 h, the plates were washed and HRP-conjugated mouse anti-human IgM mAb KT38 (Absea) diluted at 1∶1000 was added as the detection Ab. After incubating for 1 h at room temperature, the plates were washed and the color was developed for 30 min using ABTS as the substrate.

### Tandem Purification of Abs using H3_1–19_ and H3_1–19_K9me Beads

4 or 5 serum samples from each subgroup as indicated in Table 1 from aSLE patients or adult healthy controls (aHC) were randomly selected and pooled together and filtered through a 0.22 µm membrane (Product No. 4612. Pall, NY, USA). To purify the anti-H3_1–19_ Abs, 1.5 ml of the filtered serum samples were absorbed with 10 µl of the H3_1–19_ beads at 4°C for 10 h with rotation. The beads were separated by centrifugation using microcentrifuge spin columns (Product No. 69725. Pierce, Rockford, IL, USA). Then the sera were absorbed for the second time with 5 µl of the H3_1–19_ beads to ensure the complete absorption of the anti-H3_1–19_ Abs. The beads were then washed twice with PBS and 5 times with 1 M NaCl. Finally, the beads were treated twice with 50 µl of 0.1 M glycine (pH 2.5) for 10 min. The eluates were collected and immediately neutralized with 2 M Tris·Cl (pH 8.5). The sera that did not bind to the H3_1–19_ beads were used for the purification of anti-H3_1–19_K9me Abs, and the procedures were essentially the same as above.

**Table 1:** Distribution of IgG and IgM anti-H3_1–19_ and H3_1–19_K9me in SLE patients and healthy controls.

Subgroup	Pediatric samples			Adult samples		
	No. of controls	No. of patients	*P* value	No. of controls	No. of patients	*P* value
IgG(1–19^low^91^low^)IgM(1–19^high^91^high^)	18 (29.0%)	9 (14.5%)	0.050	32 (42.7%)	5 (6.7%)	<0.0001
IgG(1–19^low^91^low^)IgM(1–19^high^91^low^)	1 (1.6%)	6 (9.7%)	0.114	5 (6.7%)	5 (6.7%)	1.000
IgG(1–19^low^91^low^)IgM(1–19^low^91^high^)	23 (37.1%)	1 (1.6%)	<0.0001	14 (18.7%)	5 (6.7%)	0.027
IgG(1–19^low^91^low^)IgM(1–19^low^91^low^)	14 (22.6%)	33 (53.2%)	0.0004	20 (26.7%)	45 (60.0%)	<0.0001
IgG(1–19^high^91^x^)IgM(1–19^x^91^x^)	4 (6.4%)	10 (16.1%)	0.154	4 (5.3%)	11 (14.7%)	0.100
Others	2 (3.2%)	3 (4.8%)	–	0	4 (5.4%)	–

1–19: H3_1–19_; 91: H3_1–19_K9me. Values over or below the mean+2SD of the healthy controls were set as high or below for IgG. Values over or below the mean of the total samples were set as high or low for IgM. x: high or low reactivity.

### Western Blot Analysis

10 µl of the eluates from the H3_1–19_ or H3_1–19_K9me beads were run on gradient 5–18% SDS-PAGE gels under reducing conditions. The proteins were then transferred onto nitrocellulose membranes (Product No. HATF00010. Millipore, Billerica, MA, USA). After blocking with 5% nonfat milk for 2 h at room temperature, the membranes were cut along the 58 kDa position into two pieces. The piece with molecular weights smaller than 58 kDa was incubated with KT47, and the piece with molecular weights larger than 58 kDa was incubated with KT16 at 4°C overnight. After washing 5 times with PBST, the membranes were incubated with HRP-conjugated goat anti-mouse IgG for 1 h at room temperature. The membranes were washed and the bands were visualized using enhanced chemiluminescence (Product No. 34079. Pierce).

### The Determination of Epitope Specificity Using Competitive ELISA

96-well microtiter plates (Kelongda) were coated with peptides cross-linked to BSA in coating buffer (1 µg/ml according to the BSA concentration, 100 µl/well) at 4°C overnight. The plates were washed with PBST and blocked with PBST containing 2% BSA for 2 h at room temperature. The Abs purified from the H3_1–19_K9me beads were diluted at 1∶100 in PBST containing 2% BSA and different concentrations of lysine or ε-amine mono-methylated lysine. After incubating for 1 h at room temperature with rotation, 100 µl of the Abs was added to each well and incubated at room temperature for 1 h. KT16 and HRP-conjugated goat anti-mouse IgG were used for the detection of bound IgM as described above.

### Polyreactivity Tests

The tests were performed as described by Tiller *et al.* [26]. Briefly, 96-well microtiter plates (MaxiSorp, Product No. 449824. Nunc, Thermo Fisher Scientific, Roskilde, Denmark) were coated with 100 µl/well of dsDNA, ssDNA and lipopolysaccharide (LPS) (Product Nos. D1501, D8899, L2630. Sigma-Aldrich) in PBS at 10 µg/ml and insulin (Product No. 090-03446. Wako, Osaka, Japan) in PBS at 5 µg/ml. After incubating at 4°C overnight, the plates were washed with PBST and blocked in 2 mM EDTA/PBST (200 µl/well) for 1 h at room temperature. 1∶200 diluted eluates from the tandem purification in 2 mM EDTA/PBST were added and incubated for 2 h. The plates were washed, and 100 µl of KT47 or KT16 in 2 mM EDTA/PBST was added and incubated for 1 h at room temperature. After washing, HRP-conjugated goat anti-mouse IgG diluted in 2 mM EDTA/PBST was added. After incubating at room temperature for 1 h, the plates were blocked with 2 mM EDTA/PBST (200 µl/well) again. Color development was performed using ABTS as the substrate.

### The Reactivity of anti-H3_1–19_K9me IgM to Histones

96-well microtiter plates (Kelongda) were coated with histones (20 µg/ml, 100 µl/well) at 4°C overnight in coating buffer. After washing with PBST, the plates were blocked in 200 µl of PBST containing 2% BSA for 1 h at room temperature. 100 µl/well of the purified IgM anti-H3_1–19_K9me Abs (1∶20 dilution) that were incubated in the presence or absence of lysine or mono-methylated lysine (5 mg/ml) were added and incubated at room temperature for 1 h. KT16 was used as the primary Ab and HRP-conjugated goat anti-mouse IgG was used as the secondary Ab.

### Statistics

All of the data were analyzed using the GraphPad Prism software (Version 5.01). For data with a normal distribution, an unpaired *t* test was performed to analyze the differences between two groups and a paired *t* test was used to compare differences within a group. The Mann-Whitney test was used to compare data with a non-normal distribution. Percentages were compared using the Chi-square test or Fisher’s exact test. Pearson test was used for calculating correlation between data with normal distribution. *P*<0.05 was considered significant.

## Results

### IgG and IgM from SLE Patients and Healthy Controls have Different Reactivity Against the H3 Peptides with or without Modifications

To determine if SLE patients produced IgG or IgM against a set of synthetic peptides corresponding to the 1–19 N-terminal residues of H3 (i.e., peptides without modifications (H3_1–19_), acetylation on lysine 9 (H3_1–19_K9ac) and mono-, di-, or trimethylation on lysine 4 or lysine 9 (H3_1–19_K4me, H3_1–19_K4me2, H3_1–19_K4me3, H3_1–19_K9me, H3_1–19_K9me2 and H3_1–19_K9me3)), we performed an ELISA using sera from 62 pSLE and 75 aSLE patients. The age- and sex-matched healthy controls were tested in parallel.

**Figure 1:**
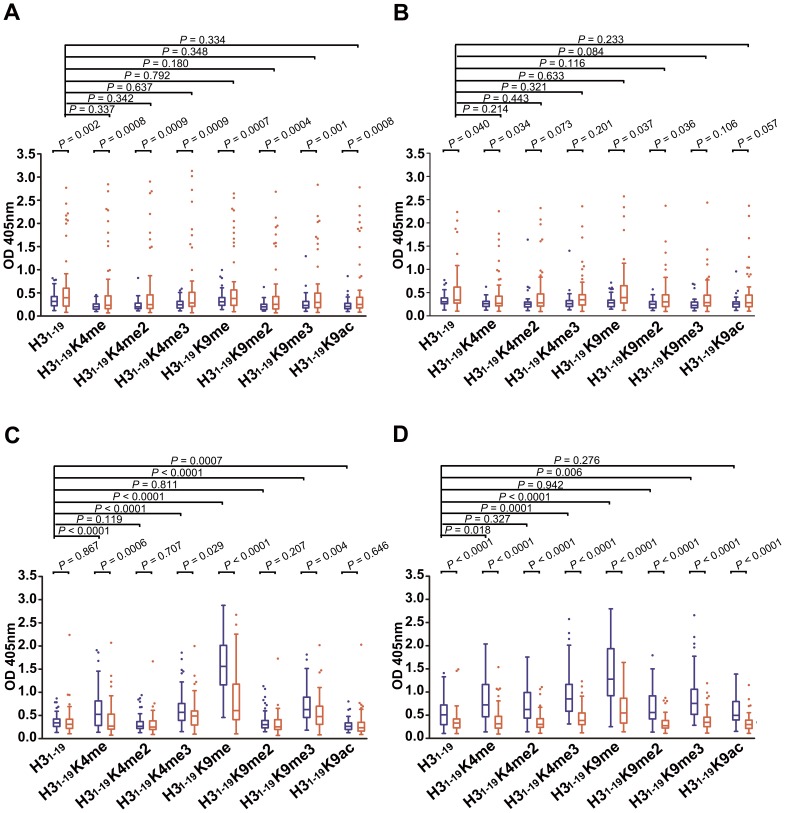
Reactivity of serum IgG and IgM against non-modified and modified H3 peptides. Microtiter plates were coated with peptides conjugated to BSA. Serum samples from healthy controls or SLE patients were diluted 1∶100 and tested. KT47 anti-human IgG and KT16 anti-human IgM mAbs were used as primary Abs, and HRP-conjugated goat anti-mouse IgG was used as a secondary Ab (see Materials and Methods). The data are presented by box-blot diagram. (A) IgG anti-peptide Abs of pediatric samples. (B) IgG anti-peptide Abs of adult samples. (C) IgM anti-peptide Abs of pediatric samples. (D) IgM anti-peptide Abs of adult samples. Healthy controls are represented in blue and SLE patients are represented in red. *P* values between the healthy control and SLE groups were calculated using the Student’s *t* test. *P* values within the same groups were calculated using the paired *t* test.

When IgG was tested, the results were as expected. Healthy subjects in general had either low levels of IgG or none at all. However, some of the SLE patients produced IgG against the peptides. There were significant differences between the pSLE patients and healthy children for all of the peptides tested (*P*≤0.002, Figure 1A). The differences were less obvious in the adult samples. Nevertheless, IgG Abs from the aSLE patients against H3_1–19_, H3_1–19_K4me, H3_1–19_K9me and H3_1–19_K9me2 were significantly higher than the levels in the healthy controls (*P*≤0.05, Figure 1B).

When IgM was tested, the results unexpectedly showed very different patterns from the IgG results. For the pediatric samples, the majority of the healthy controls reacted strongly to H3_1–19_K9me and moderately to H3_1–19_K4me, H3_1–19_K4me3 and H3_1–19_K9me3 (Figure 1C). In comparison, the pSLE patients surprisingly had lower levels of the IgM Abs against H3_1–19_K9me (*P<*0.0001), although these levels were still the highest among all of the peptides tested. IgM reactivity in the pSLE patients against H3_1–19_K4me, H3_1–19_K4me3 and H3_1–19_K9me3 was also lower than the levels in the healthy controls (*P*≤0.029). Similar results occurred for the adult samples, except that the IgM Abs against H3_1–19_K4me2, H3_1–19_K9me2 and H3_1–19_K9ac from the aHC were also higher than the IgM levels in the aSLE patients (Figure 1D).

**Figure 2:**
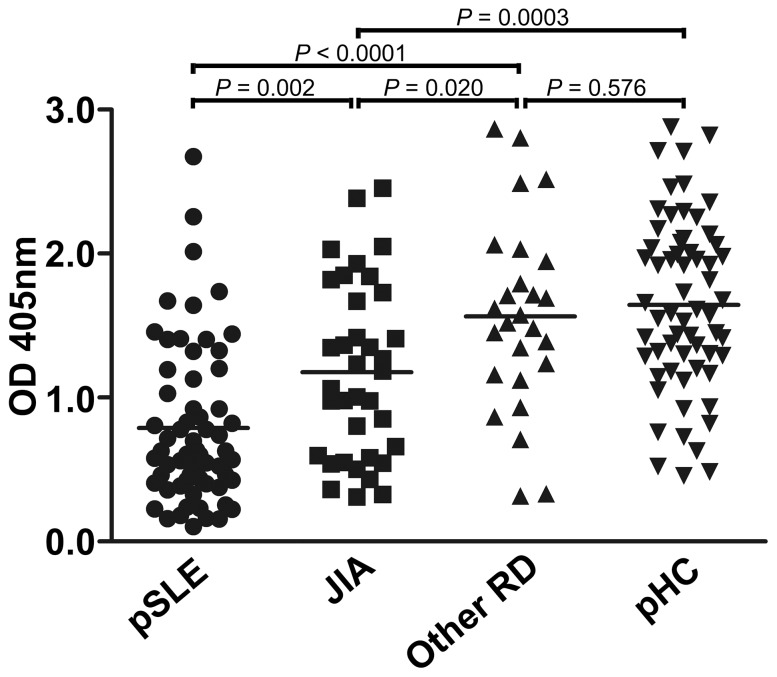
Comparison of IgM anti-H3_1–19_K9me levels in patients and healthy subjects. Anti-H3_1–19_K9me IgM levels in pSLE patients (n = 62), JIA patients (n = 36), other RD patients (n = 26, including juvenile ankylosing spondylitis, Henoch-Schonlein purpura, idiopathic thrombocytopenic purpura, Kawasaki disease, mixed connective tissue disease, juvenile dermatomyositis and Behcet’s disease) and pHC (n = 62) were measured by ELISA. Microtiter plates were coated with the H3_1–19_K9me peptide conjugated to BSA. The serum samples were diluted 1∶100 and tested. The KT16 anti-human IgM mAb was used as the primary Ab, and HRP-conjugated goat anti-mouse IgG was used as the secondary Ab. The horizontal bars indicate the mean values in each group. *P* values were calculated using the Student’s *t* test.

We also measured IgM anti-H3_1–19_K9me in JIA patients and other RD patients, including juvenile ankylosing spondylitis, Henoch-Schonlein purpura, idiopathic thrombocytopenic purpura, Kawasaki disease, mixed connective tissue disease, juvenile dermatomyositis and Behcet’s disease. The IgM levels in the JIA patients were also lower than the levels in the healthy controls. Nevertheless, the levels were still significantly higher than the levels in the pSLE patients (Figure 2). There was no significant difference between other RD patients and the healthy controls.

### IgG from SLE Patients and IgM from Healthy Controls have Different Epitope Preferences for H3_1–19_ and H3_1–19_K9me

All of the peptides tested had an identical H3 N-terminal sequence, ARTKQTARKSTGGKAPRKQ. Differences between the peptides only appeared at the positions of the modified amino acids. Therefore, these peptides had shared epitopes that could be recognized by cross-reactions. If such cross-reactions were dominant, the Abs against the non-modified and modified peptides would show similar results. Based on this assumption, we performed a paired *t* test to compare the reactivity of the Ab against H3_1–19_ and each of the modified peptides. We found that there was no significant difference between IgG against H3_1–19_ and any of the modified peptides in the SLE patients (*P*≥0.084, Figure 1, A and B)_,_ suggesting that the IgG Abs were mainly against the shared epitopes on the peptides. In contrast, there were significant differences between IgM against H3_1–19_ and IgM against the modified peptides (H3_1–19_K4me, H3_1–19_K4me3, H3_1–19_K9me, H3_1–19_K9me3) (*P*≤0.018, Figure 1, C and D) in both the pediatric healthy controls (pHC) and aHC, suggesting that IgM could specifically recognize epitopes that were only on the modified peptides.

These results suggest that IgM and IgG might have different epitope preferences for the peptides. However, this is unclear because the raw serum samples would inevitably result in non- or low-specific signals. Therefore, we used affinity purified Abs to test the specificities. We were only able to purify adult Abs against H3_1–19_ and H3_1–19_K9me due to sample availability. The samples were divided into five subgroups according to their reactivity against the peptides. Values for IgG over the mean+2SD of the healthy controls were set as high, and values for IgM over the mean of the total samples were set as high. Thus the five subgroups were IgG(1–19^low^91^low^)IgM(1–19^high^91^high^), IgG(1–19^low^91^low^)IgM(1–19^high^91^low^), IgG(1–19^low^91^low^)IgM(1–19^low^91^high^), IgG(1–19^low^91^low^)IgM(1–19^low^91^low^) and IgG(1–19^high^91^x^)IgM(1–19^x^91^x^) (Table 1). 1–19 represents peptide H3_1–19_ and 91 represents peptide H3_1–19_K9me, respectively. The subgroup IgG(1–19^high^91^x^)IgM(1–19^x^91^x^) had high levels of IgG reactivity against H3_1–19_ but varied IgG reactivity against H3_1–19_K9me and varied IgM reactivity against both peptides. These five subgroups included samples from100% of the healthy adults and 95% of the aSLE patients.

For Ab purification, 4 or 5 serum samples within a subgroup were pooled together. Equal volumes (1.5 ml) of the pooled sera were absorbed twice with H3_1–19_ beads. The unbound Abs in the sera were then absorbed with H3_1–19_K9me beads. The beads were washed with 1 M NaCl to remove the non- or low- specific Abs bound on the beads. The Abs were then separately eluted and analyzed by Western blot and ELISA.

**Figure 3:**
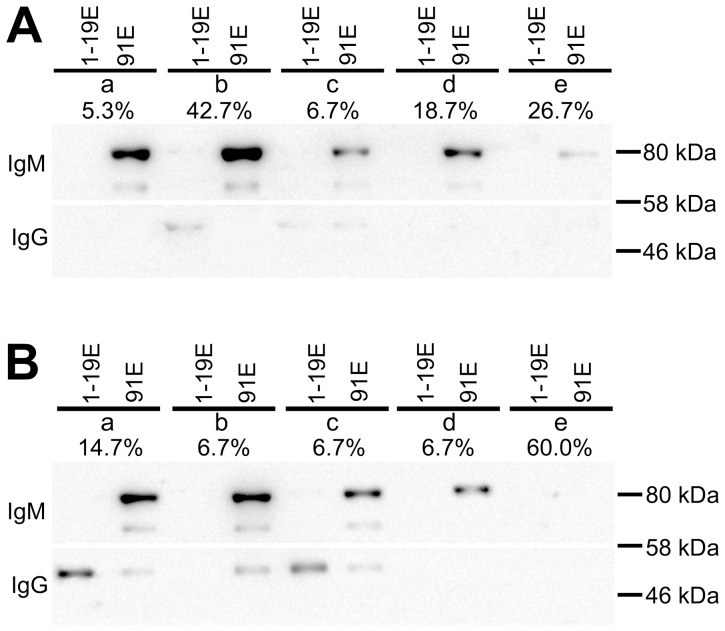
Western blot for IgG and IgM eluted from H3_1__–19_ and H3_1–19_K9me beads. Equal volumes (1.5 ml) of pooled sera were first absorbed with the H3_1–19_ beads and then with the H3_1–19_K9me beads as described in the Materials and Methods. The beads were washed 5 times with 1 M NaCl, and the bound Abs were eluted with 50 µl elution buffer. 10 µl of the eluates underwent SDS-PAGE under reducing conditions and were then transferred onto nitrocellulose membranes. The membranes were cut at the 58 kDa position. IgG on the low molecular weight (<58 kDa) membrane and IgM on the high molecular weight (>58 kDa) membrane were detected. (A) Healthy adult samples; (B) aSLE patient samples. a, IgG(1–19^high^91^x^)IgM(1–19^x^91^x^); b, IgG(1–19^low^91^low^)IgM(1–19^high^91^high^); c, IgG(1–19^low^91^low^)IgM(1–19^high^91^low^); d, IgG(1–19^low^91^low^)IgM(1–19^low^91^high^); e, IgG(1–19^low^91^low^)IgM(1–19^low^91^low^). 1–19E, Abs eluted from the H3_1–19_ beads; 91E, Abs eluted from the H3_1–19_K9me beads. The percentages represent the sample proportion of each subgroup (see Table 1). The results are representative of two independent experiments.

Western blot was performed to detect the Ab isotypes and amounts of IgG and IgM in the eluates from each subgroup. The results are shown in Figure 3. For the Abs purified using the H3_1–19_ beads, IgG was barely detectable in the eluates from the healthy controls. However, the IgG Abs were clearly detectable in the IgG(1–19^high^91^x^)IgM(1–19^x^91^x^) subgroup of aSLE patients. IgG Abs were also detectable in the IgG(1–19^low^91^low^)IgM(1–19^high^91^low^) subgroup, which was categorized as low IgG reactivity against H3_1–19_. IgM was undetectable in both the SLE and healthy controls, including the IgG(1–19^low^91^low^)IgM(1–19^high^91^high^), IgG(1–19^low^91^low^)IgM(1–19^high^91^low^) and IgG(1–19^high^91^x^)IgM(1–19^x^91^x^) subgroups, indicating that the high IgM reactivity in Figure 1 was due to low- or non-specific binding.

For the Abs purified using the H3_1–19_K9me beads, a heavy IgM band, but not IgG band, appeared in the IgG(1–19^low^91^low^)IgM(1–19^high^91^high^) subgroup of the healthy controls (Figure 3A), which represented 42.7% of the healthy samples. IgM could also be detected in all of the other subgroups of the healthy controls, including IgG(1–19^low^91^low^)IgM(1–19^high^91^low^) and IgG(1–19^low^91^low^)IgM(1–19^low^91^low^), which were categorized as low IgM reactivity against H3_1–19_K9me. In comparison, anti-H3_1–19_K9me IgM was detectable in all the subgroups of the SLE patients except for the IgG(1–19^low^91^low^)IgM(1–19^low^91^low^) subgroup, but in lower quantities. No IgM could be detected in the IgG(1–19^low^91^low^)IgM(1–19^low^91^low^) subgroup, which represented 60% of the patient samples. IgG could also be detected in the H3_1–19_K9me eluates from the IgG(1–19^high^91^x^)IgM(1–19^x^91^x^), IgG(1–19^low^91^low^)IgM(1–19^high^91^high^) and IgG(1–19^low^91^low^)IgM(1–19^high^91^low^) subgroups, although in lower quantities.

**Figure 4:**
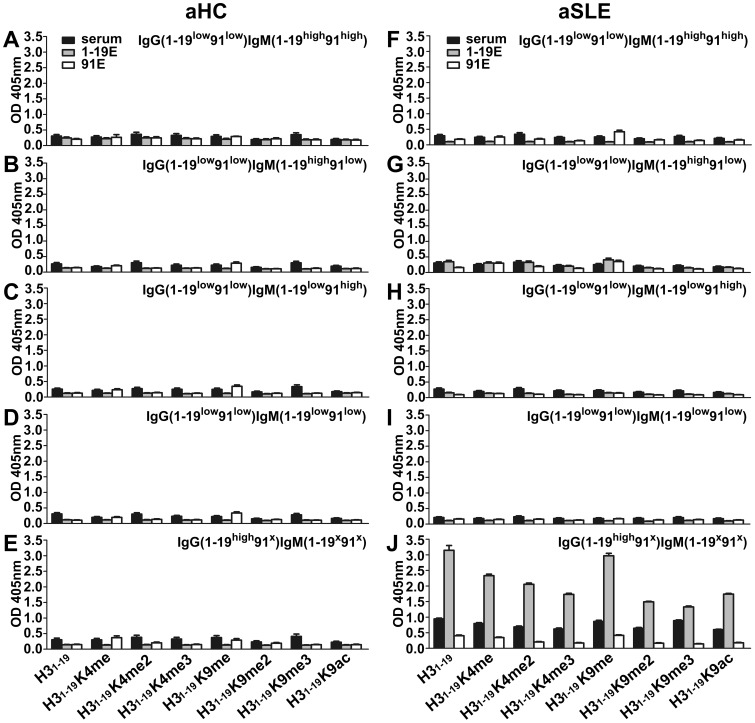
Reactivity of purified IgG against H3 peptides. Equal volumes (1.5 ml) of the pooled serum samples from each subgroup were affinity purified using H3_1–19_ and H3_1–19_K9me beads in tandem (see Materials and Methods). Microtiter plates were coated with various peptides conjugated to BSA as indicated. Eluates from the H3_1–19_ and H3_1–19_K9me beads were separately tested for their reactivity against the peptides. KT47 anti-human IgG was used as the primary Ab and HRP-conjugated goat anti-mouse IgG was used as the secondary Ab. The samples were tested in duplicate. 1–19E, Abs eluted from the H3_1–19_ beads; 91E, Abs eluted from the H3_1–19_K9me beads. Data are expressed as mean+SEM. The results are representative of two independent experiments.

**Figure 5:**
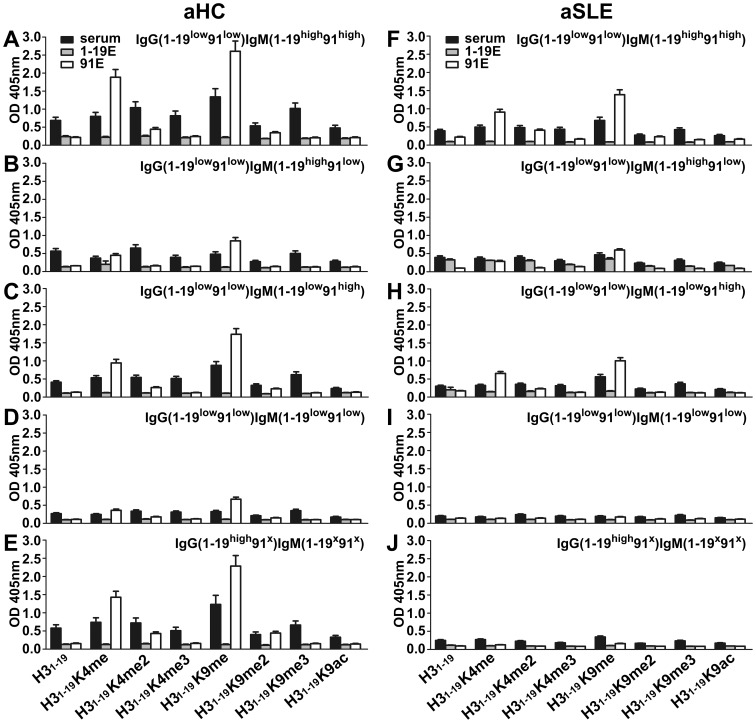
Reactivity of purified IgM against H3 peptides. Equal volumes (1.5 ml) of the pooled serum samples from each subgroup were affinity purified using H3_1–19_ and H3_1–19_K9me beads in tandem (see Materials and Methods). Microtiter plates were coated with various peptides conjugated to BSA as indicated. Eluates from the H3_1–19_ and H3_1–19_K9me beads were separately tested for their reactivity against the peptides. KT16 anti-human IgM was used as the primary Ab and HRP-conjugated goat anti-mouse IgG was used as the secondary Ab. The samples were tested in duplicate. 1–19E, Abs eluted from the H3_1–19_ beads; 91E, Abs eluted from the H3_1–19_K9me beads. Data are expressed as mean+SEM. The results are representative of two independent experiments.

An ELISA was performed to identify the Ab specificities against each of the peptides. The results are shown in Figure 4 and 5. In the healthy controls, there was IgG from neither the H3_1–19_ eluates nor the H3_1–19_K9me eluates (Figure 4, A–E). In comparison, IgM Abs from the H3_1–19_K9me eluates were clearly detectable in the IgG(1–19^low^91^low^)IgM(1–19^high^91^high^), IgG(1–19^low^91^low^)IgM(1–19^low^91^high^) and IgG(1–19^high^91^x^)IgM(1–19^x^91^x^) subgroups (Figure 5, A, C, and E) subgroups, which represented 67% of the aHC. IgM Abs from the H3_1–19_K9me eluates were even detectable in the IgG(1–19^low^91^low^)IgM(1–19^high^91^low^) and IgG(1–19^low^91^low^)IgM(1–19^low^91^low^) subgroups (Figure 5, B and D), which were categorized as low IgM reactivity in the serum tests. Interestingly, IgM from the H3_1–19_K9me eluate reacted to H3_1–19_K4me but not to the other peptides (Figure 5, A, C, and E). The results from the aSLE patients were more complex. For the H3_1–19_ eluates, IgG Abs in the IgG(1–19^high^91^x^)IgM(1–19^x^91^x^) subgroup, but not the other subgroups, were detectable and able to cross-react with all of the peptides (Figure 4J), indicating that these Abs recognized shared epitopes on the peptides. For the H3_1–19_K9me eluates, IgG Abs were barely detectable in any of the SLE subgroups (Figure 4, F–J), but IgM could be detected in the IgG(1–19^low^91^low^)IgM(1–19^high^91^high^) and IgG(1–19^low^91^low^)IgM(1–19^low^91^high^) subgroups, which represented 13.3% of aSLE patients, although the signals were weaker (Figure 5, F and H). Similar to the healthy controls, IgM cross-reacted to H3_1–19_K4me but not to the other peptides.

### The Epitope for IgM Anti-H3_1–19_K9me is a Mono-methylated Lysine

**Figure 6:**
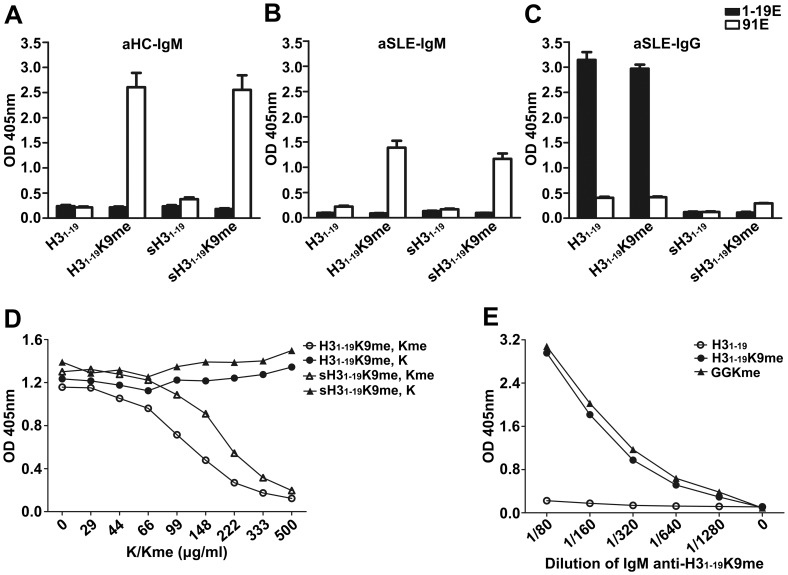
Epitope analysis for IgG and IgM purified from the H3_1–19_ or H3_1–19_K9me beads. (A), (B) and (C) Microtiter plates were coated with H3_1–19_, H3_1–19_K9me, scrambled H3_1–19_ (sH3_1–19_) and scrambled H3_1–19_K9me (sH3_1–19_K9me) conjugated to BSA. IgM of healthy adults from the IgG(1–19^low^91^low^)IgM(1–19^high^91^high^) subgroup, IgM of aSLE patients from the IgG(1–19^low^91^low^)IgM(1–19^high^91^high^) subgroup and IgG of aSLE patients from the IgG(1–19^high^91^x^)IgM(1–19^x^91^x^) subgroup were tested. KT16 anti-human IgM mAb and KT47 anti-human IgG were used as the primary Abs. HRP-conjugated goat anti-mouse IgG was used as the secondary Ab. 1–19E, Abs eluted from the H3_1–19_ beads; 91E, Abs eluted from the H3_1–19_K9me beads. Data are expressed as mean+SEM. (D) Microtiter plates were coated with H3_1–19_K9me or sH3_1–19_K9me conjugated to BSA. IgM Abs of healthy adults from the IgG(1–19^low^91^low^)IgM(1–19^high^91^high^) subgroup were incubated with lysine (K) or ε-amine mono-methylated lysine (Kme) at indicated concentrations for 1 h at room temperature. KT16 anti-human IgM was used as the primary Ab. HRP-conjugated goat anti-mouse IgG was used as the secondary Ab. (E) Microtiter plates were coated with H3_1–19_, H3_1–19_K9me and GGK(me)GGSGGSGGSG (GGKme) conjugated to BSA. IgM of healthy adults from the IgG(1–19^low^91^low^)IgM(1–19^high^91^high^) subgroup was tested. KT16 anti-human IgM was used as the primary Ab. HRP-conjugated goat anti-mouse IgG was used as the secondary Ab. The results are representative of three separate experiments.

As mentioned above, IgM purified from the H3_1–19_K9me beads cross-reacted with H3_1–19_K4me but not the other peptides. To determine if the Abs recognized special sequences on the H3_1–19_K4me and H3_1–19_K9me epitopes, a scrambled peptide, KTRARAQTK(me)TGSAGKQKPR, was tested. This peptide contained the same amino acid composition but a different sequence as H3_1–19_K9me. Our results indicated that IgM from healthy adults and aSLE patients purified using the H3_1–19_K9me beads reacted to the scrambled peptide (Figure 6, A and B). In contrast, IgG from aSLE patients purified using the H3_1–19_ beads could not recognize the scrambled H3_1–19_, KTRARAQTKTGSAGKQKPR (Figure 6C), indicating that the IgG Abs were against epitopes with fixed sequences. Furthermore, ε-amine mono-methylated lysine could completely inhibit IgM binding to H3_1–19_K9me, but lysine had no inhibitory effect (Figure 6D). Finally, the IgM Abs could similarly bind an artificial peptide, GGK(me)GGSGGSGGSG (GGKme) (Figure 6E). Thus, mono-methylated lysine was the sole epitope for IgM.

The 25 raw serum samples randomly selected from aSLE patients and adult controls also reacted against GGKme, and the differences between the aSLE and control sera were more obvious because the serum may have contained less non-specific binding to GGKme than to H3_1–19_K9me (Figure S2).

It can be noted that IgM binding to H3_1–19_K4me was weaker than IgM binding to H3_1–19_K9me, which was not due to the lower affinity of the Abs, but rather to less quantities of H3_1–19_K4me coated on the plates, as H3_1–19_K4me had a lower degree of cross-linking than H3_1–19_K9me to BSA (Figure S1).

### IgM and IgG Against H3_1–19_ and H3_1–19_K9me are not Polyreactive

**Figure 7:**
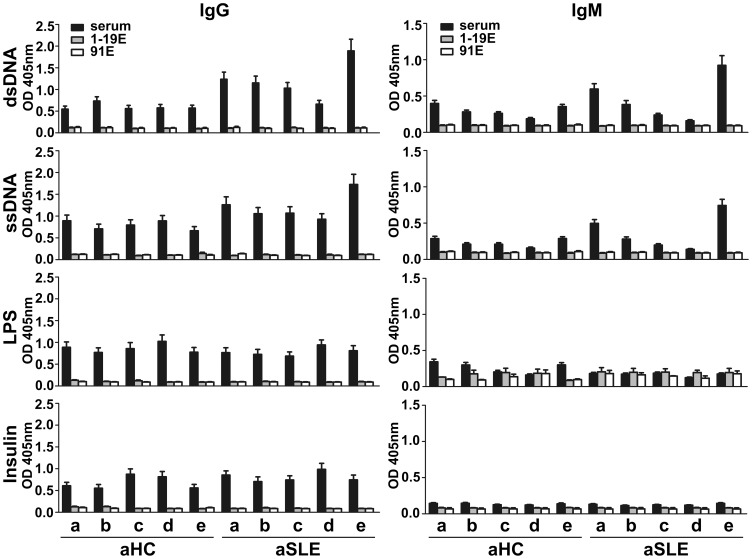
Polyreactivity test by ELISA. dsDNA, ssDNA, LPS or insulin was coated on microtiter plates as described in Materials and Methods. Sera (1∶100 dilution) and Abs purified from the H3_1–19_ or H3_1–19_K9me beads (1∶200 dilution) were tested. KT47 anti-human IgG or KT16 anti-human IgM Ab was used as the primary Ab and HRP-conjugated goat anti-mouse IgG was used as the secondary Ab. a, IgG(1–19^low^91^low^)IgM(1–19^high^91^high^); b, IgG(1–19^low^91^low^)IgM(1–19^high^91^low^); c, IgG(1–19^low^91^low^)IgM(1–19^low^91^high^); d, IgG(1–19^low^91^low^)IgM(1–19^low^91^low^); e, IgG(1–19^high^91^x^)IgM(1–19^x^91^x^). 1–19E, Abs eluted from the H3_1–19_ beads; 91E, Abs eluted from the H3_1–19_K9me beads. Data are expressed as mean+SEM. The results are representative of two separate experiments.

**Figure 8:**
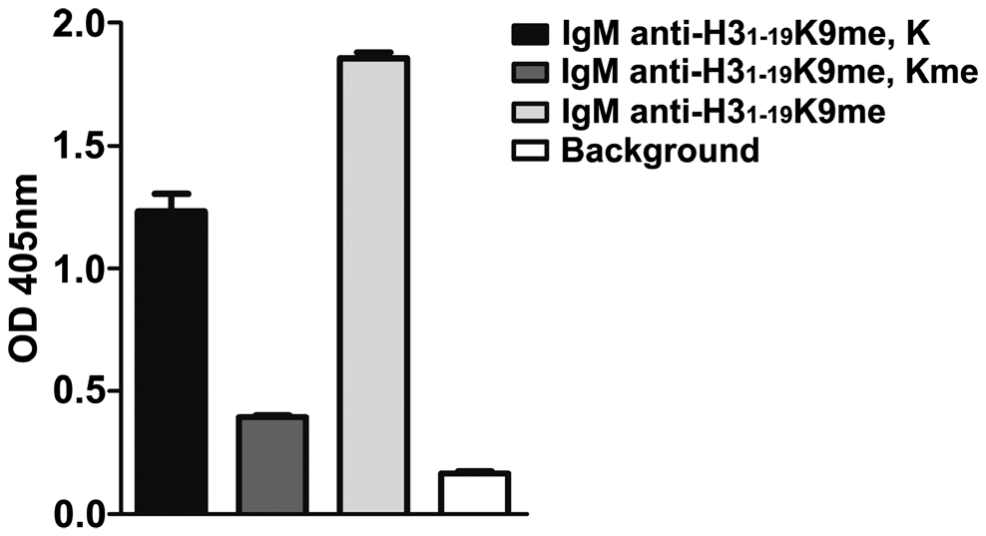
Reactivity of anti-H3_1–19_K9me IgM against histones. Microtiter plates were coated with histones. IgM Abs purified from the H3_1–19_K9me beads with or without prior absorption with lysine or mono-methylated lysine were tested. Background wells were not added with purified IgM. KT16 anti-human IgM was used as the primary Ab and HRP-conjugated goat anti-mouse IgG was used as the secondary Ab. Data are expressed as mean+SEM. The results are representative of three separate experiments.

To determine if the purified IgG and IgM Abs were polyreactive to other antigens, we tested these Abs against dsDNA, ssDNA, LPS and insulin. No reactivity was observed for either IgG or IgM purified using the H3_1–19_ or H3_1–19_K9me beads (Figure 7). However, IgM recognized histones extracted from Hela cells (Figure 8). This reaction was weak, presumably because either the histone lysine residues were not heavily mono-methylated or the modification was not properly exposed. Nevertheless, mono-methylated lysine was able to inhibit the reaction, indicating that the reaction between IgM and the histones was specific.

### IgM Anti-H3_1–19_K9me Abs do not Exist at Birth

**Figure 9:**
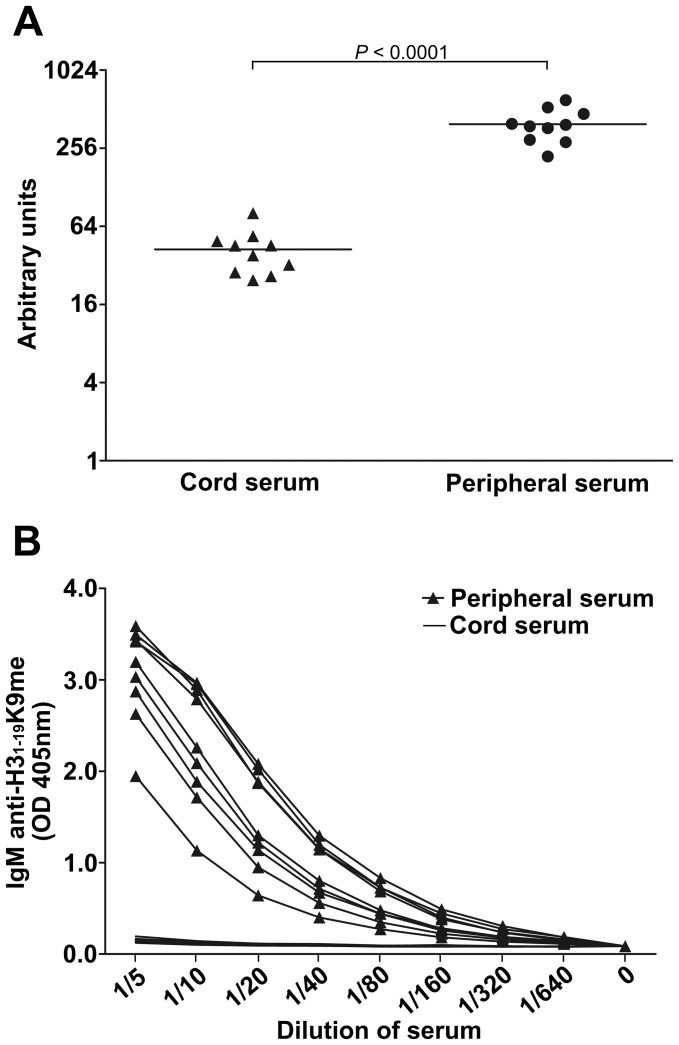
Comparison of anti-H3_1–19_K9me IgM in sera from peripheral blood and umbilical cord blood. (A) Comparison of total IgM concentration in sera. KT16 anti-human IgM was used as the capture Ab and HRP-conjugated KT38 was used as the detection Ab. Concentrations of IgM are represented as arbitrary units calculated using a serum titration curve. The horizontal bars indicate the mean values in each group. *P* value was calculated using Student’s *t* test. (B) Anti-H3_1–19_K9me IgM in 9 peripheral serum samples from healthy children or in 10 cord serum samples. Microtiter plates were coated with H3_1–19_K9me peptides conjugated to BSA. KT16 anti-human IgM was used as the primary Ab and HRP-conjugated goat anti-mouse IgG was used as the secondary Ab. The results are representative of three separate experiments.

To determine if the IgM anti-H3_1–19_K9me Abs were formed against self-antigens, we collected 10 umbilical cord sera samples and compared them with IgM from the peripheral blood. The IgM concentrations in the cord blood were only about one tenth of the concentrations in the peripheral blood (Figure 9A). Considering the concentration differences, we tested the serum samples in serial dilutions. As shown in Figure 9B, all of the IgM from the peripheral blood were reactive against H3_1––19_K9me, but none of the cord IgM reacted with the peptide, indicating that IgM against H3_1–19_K9me was not generated to react with self-antigens.

### Levels of IgG Anti-H3_1–19_ and IgM Anti-H3_1–19_K9me Abs are not Correlated with Disease Activity

**Figure 10:**
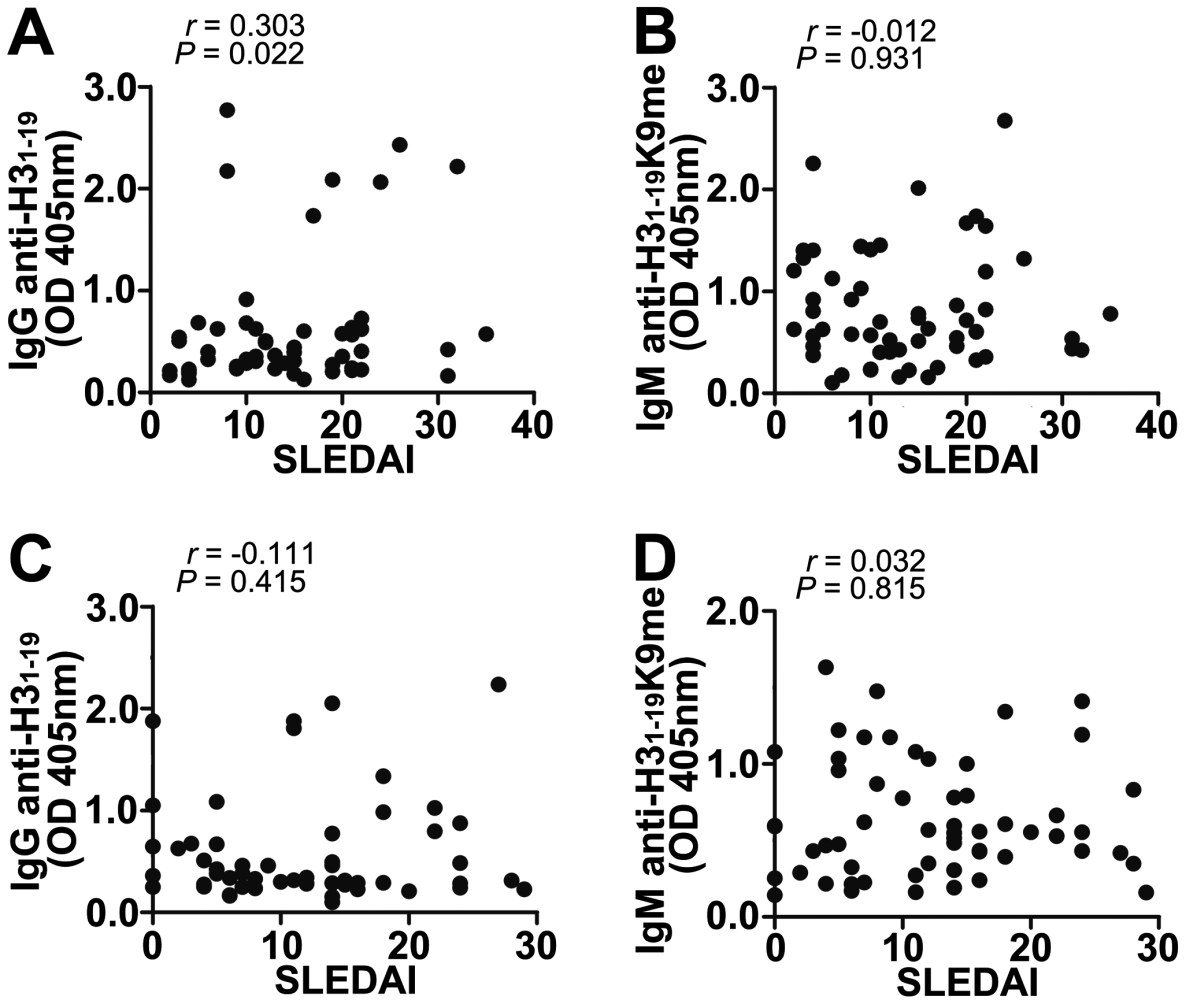
Correlations between SLEDAI scores and levels of IgG anti-H3_1–19_, IgM anti-H3_1–19_K9me. (A) Correlation between IgG anti-H3_1–19_ and SLEDAI of pSLE. (B) Correlation between IgM anti-H3_1–19_ K9me and SLEDAI of pSLE. (C) Correlation between IgG anti-H3_1–19_ and SLEDAI of aSLE. (D) Correlation between IgM anti-H3_1–19_K9me and SLEDAI of aSLE. The correlations were analyzed by Pearson correlation analysis. pSLE: n = 57, male/female = 11/46, age 10.87±2.95 yrs old; aSLE: n = 54, male/female = 10/44, age 33.00±11.00 yrs old.

We studied correlations between IgG anti-H3_1–19_ and IgM anti-H3_1–19_K9me with disease activity indicated by SLE disease activity index (SLEDAI). No significant correlations were found except for IgG in the pediatric patients, which was weakly correlated with SLEDAI (*r* = 0.303, Figure 10).

### The Low IgM Activity of SLE Patients against H3_1–19_K9me is not caused by Medical Treatments

**Figure 11:**
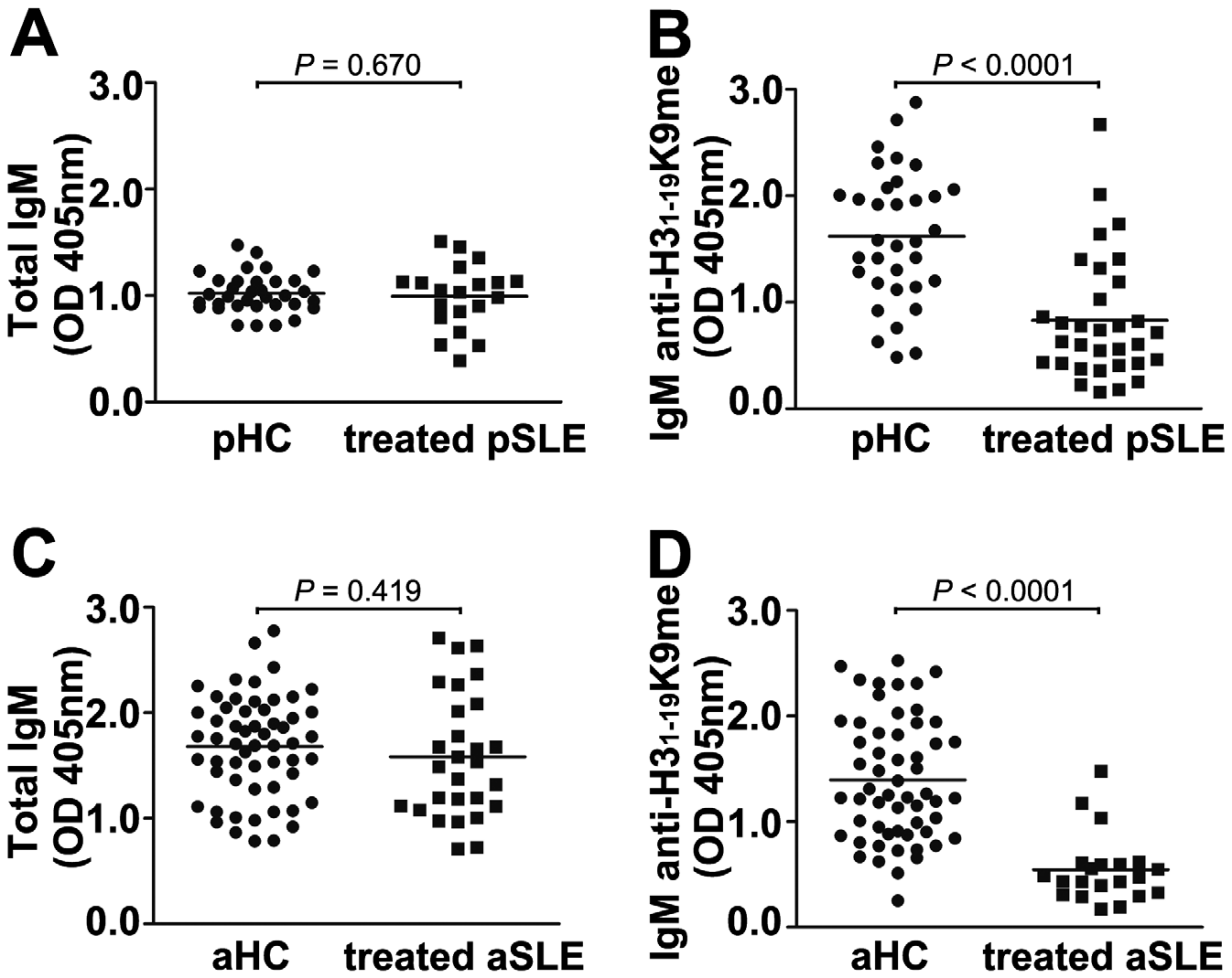
Effect of medical treatments on total IgM and IgM anti-H3_1–19_K9me. (A) and (C) Total IgM of pediatric and adult samples. (B) and (D) IgM anti-H3_1–19_K9me of pediatric and adult samples. *P* values between the healthy controls and treated SLE were calculated using the Student’s *t* test. The horizontal bars indicate the mean values in each group. The results are representative of two independent experiments.

Some of the patients were under medical treatments (Table S2) when their blood was collected. To see if the treatments reduced total IgM levels and thus caused the low IgM activity against H3_1–19_K9me, we compared total IgM of patients with that from normal controls. There was no significant difference between them (Figure 11, A and C). The activity of IgM anti-H3_1–19_K9me of healthy controls was still significantly high than that of patient groups (Figure 11, B and D).

## Discussion

In this work, we measured the reactivity of IgG and IgM from SLE patients and healthy subjects against a set of H3 N-terminal peptides with or without methyl or acetyl modifications. We found that some SLE patients, but not healthy subjects, produced IgG against the non-modified peptide, which is in agreement with other studies [8]. IgG also reacted with the modified peptides, but this reactivity was due to cross-reactions on the shared epitopes between H3_1–19_ and the modified peptides. Importantly, we report that the majority of the healthy subjects produced IgM against mono-methylated lysine with considerably high titers, and the levels of these IgM Abs in SLE patients were substantially lower than the levels in the healthy controls.

It was surprising to observe that IgM were specifically produced against an epitope with only one amino acid, i.e., mono methylated lysine. It is known that poly−/auto-reactive IgM Abs play important roles in the protection against autoimmune diseases [27], [28]. Genetically manipulated mice that were deficient in secretory IgM but not other Ab classes had an increased propensity to the spontaneous development of IgG anti-DNA Abs and the renal deposition of IgG and complement [29], [30]. Lupus-prone mice treated with murine IgM anti-dsDNA Abs exhibited a delayed onset of proteinuria and a reduced degree of renal pathology, which resulted in significantly improved survival [31]. The expression of ppc1–5, a natural IgM autoAb, in MRL-lpr mice prevented proteinuria and reduced kidney immune complex formation [32]. MRL/lpr mice deficient in activation-induced deaminase produced high levels of autoreactive IgM but lacked autoreactive IgG. These mice showed a significant reduction in glomerulonephritis and a dramatic increase in survival [33]. In humans, it is known that IgG Abs against dsDNA are involved in the pathogenesis of SLE glomerulonephritis, but this disease is rare in SLE patients with IgM against dsDNA. There was a negative correlation between anti-dsDNA IgM Abs and glomerulonephritis [34]. SLE patients with low disease activity tended to have higher levels of polyreactive IgM Abs [35]. Anti-phosphorylcholine IgM was significantly higher in patients with low disease activity and less organ damage, and anti-cardiolipin and anti-dsDNA IgM were significantly higher in patients without renal disease [36]. IgM against mono-methylated lysine found in this work could be classed as an autoAb because mono-methylated lysine is present on histones in eukaryotic cells [37]. Other proteins, such as p53, NFκB and STAT3, also have mono-methylated lysine [38]. Although we presently do not know the exact effector functions of these Abs, the fact that healthy subjects have significantly higher levels of these Abs than SLE patients suggests the possibility that these Abs are required for healthy conditions, and reduced levels may lead to SLE (-like) autoimmune diseases.

Several mechanisms have been elucidated for IgM autoAbs in preventing autoimmune diseases. IgM autoAbs are efficient in binding and neutralizing autoantigens like dsDNA. The immune complexes are phagocytosed rather than deposited on the glomerular basement membrane [31]. IgM autoAbs facilitate the removal of apoptotic cells, and thus the reduction of IgM may result in an impaired clearance of apoptotic cells and cell debris, which may stimulate the production of pathogenic IgG autoAbs. Although the functions of IgM against mono-methylated lysine are not known, it is possible that histones are the major targets of these Abs, as histones are the most abundant proteins associated with lysine methylation, and only a few methylated non-histone proteins have been found [38]. Because it is difficult for IgM to penetrate cells, the possible effector sites for IgM Abs against mono-methylated lysine would be on the cell surface or with cell debris, where the Abs could bind histones released from damaged or apoptotic cells and subsequently activate complement to facilitate the removal of unwanted histones.

Autoreactive IgM can be divided into natural or immune Abs. The natural autoAbs (NAA) are produced without exogenous antigen stimulation, as they are present in the cord blood from newborn humans and mice as well as in mice housed in germ-free conditions and fed an antigen-free diet [32]. B1 cells are the major source of NAA, but B2 cells, including marginal zone and follicular B cells, can also produce NAA [27], [32], [39]. The origin of IgM Abs against mono-methylated lysine is not presently known. It was originally thought these Abs were NAA produced against self-mono-methylated lysine on histones or other proteins. If this was the case, these IgM should have been produced before birth in the absence of foreign antigens. However, we did not observe any IgM Abs against mono-methylated lysine in the umbilical cord sera where substantial amounts of IgM exist (Figure 9). In addition, the Abs did not react against dsDNA, ssDNA, LPS and insulin, which is the general property of polyreactive NAA [40]. Furthermore, most NAA have low affinities to their antigens, but the IgM Abs bound mono-methylated lysine with high affinity, as washing with1 M NaCl did not disassociate these Abs from the H3_1–19_K9me beads. Therefore, these IgM might be ‘immune’ Abs which are acquired in response to foreign antigens after birth and somehow gain the ability to react against self-mono-methylated lysine through mechanisms such as molecular mimicry [41]. As histone methylation is very common in eukaryotic cells, humans can easily access mono-methylated lysine from countless microorganisms through various infections [42]. In this study, IgM could be detected in children as early as 2 yrs old. It would be interesting to determine when babies start to produce these Abs. To determine whether the Abs against mono-methylated lysine exist in other species, we measured IgM against mono-methylated lysine in BALB/c and C57BL/6 mice, but the levels were undetectable (unpublished observations). This may be because the animals were kept in specific pathogen free conditions where they were unable to contact the necessary antigens to evoke a response against the mono-methylated lysine.

Both the healthy subjects and patients with autoimmune diseases produced autoAbs. However, the relationship between the autoAbs produced in the healthy subjects (mostly the IgM isotype) and the pathogenic autoAbs produced in the patients (mostly the IgG isotype) is still unclear. It is speculated that natural poly−/auto-reactive Abs may occasionally promote autoimmunity by serving as a template for the high affinity pathogenic autoAbs. These poly−/auto-reactive Abs may also suppress autoimmunity by regulating excessive autoimmune and inflammatory responses [32]. It is unknown if the autoreactive IgM produced in the healthy status is a major source of pathologic IgG autoAbs in disease or if the autoAbs in disease have different origins. Although NAA are mainly produced by B1 cells, there is little evidence in humans that B1 cells are the sources of pathogenic autoreactivity, although there are murine models of systemic lupus where B1 cells are the source of pathogenic autoAbs [43]. However, cells of the B2 origin, including marginal zone and follicular B cells, are more likely linked to pathologic autoAbs. An analysis of healthy people reported that about 40% of the new emigrant B cells from the bone marrow (mainly of the B2 subset) and about 20% of the mature naïve B cells had features of autoreactivity and reacted to HEp-2 cells [44]. Although most of these cells will not go into the plasma cell pools, they have the potential to become autoAb secreting cells. In addition, somatic hypermutation creates autoreactive IgG^+^ memory B cells [45]. It has been observed that SLE patients are defective in removing autoreactive B cells from new emigrant B cells, and these cells may have acquired autoreactivity from somatic hypermutations [46]–[48]. In our study, we were unable to find any links between anti-H3_1–19_ IgG and anti-Kme IgM, although both of these Abs recognize the H3 N-terminal peptides. We did observe that SLE patients produced little but detectable anti-H3_1–19_K9me IgG (Figure 3). However, we do not know whether they were from anti-mono-methylated lysine IgM by class switching or from a different origin.

In conclusion, we have identified mono-methylated lysine as a novel epitope for IgM autoAbs in a majority of healthy subjects, and the levels of these IgM Abs in SLE patients are significantly lower than that in the healthy controls. These IgM autoAbs are acquired after birth and can recognize peptides or proteins containing mono-methylated lysine residues but not di- or tri-methylated lysine. Although the serum IgM levels against mono-methylated lysine do not associate with disease activity, they may still be useful in discriminating SLE patients from healthy persons.

## Supporting Information

Figure S1 **SDS-PAGE of H3 peptides cross-linked to BSA.** Synthetic peptide was cross-linked onto BSA. 2 µg (according to the BSA concentration) of each peptide/BSA run on 10% SDS-PAGE under reducing conditions.
(TIF)

Figure S2 **Serum reactivity to H3_1–19_K9me and GGKme.** Microtiter plates were coated with H3_1–19_K9me and GGKme conjugated to BSA. Serum samples (n = 25) from the healthy adults or aSLE patients were diluted 1∶100 and tested. KT16 anti-human IgM was used as the primary Ab and HRP-conjugated goat anti-mouse IgG was used as the secondary Ab. Data are expressed as mean±SEM. (A) H3_1–19_K9me coated. (B) GGKme coated. The results are shown as representative of two separate experiments.
(TIF)

Table S1 **Information about the SLE patients and healthy controls.** a, M, male; F, female. Ratios were compared by Chi-square test. b, Ages were compared by Mann-Whitney test. *P*<0.05 was considered significant.
(DOCX)

Table S2 **Treatments received by SLE patients at blood collection.** GC, Glucocorticoids; IS, Immunosuppressants; AM, Antimalarials; mAbs, monoclonal antibodies; IVIG, Intravenous immunoglobin. a, The number of patients treated with/without that medicine.
(DOCX)

Table S3 **Peptides and their modifications.**
(DOCX)
