# Supplementary figures and images for: Systemic Lupus Erythematosus Patients Contain Significantly Less IgM against Mono-Methylated Lysine than Healthy Subjects
Source: PLoS One. 2013 Jul 16;8(7):e68520. doi: 10.1371/journal.pone.0068520 (PMC3713014; doi:10.1371/journal.pone.0068520)

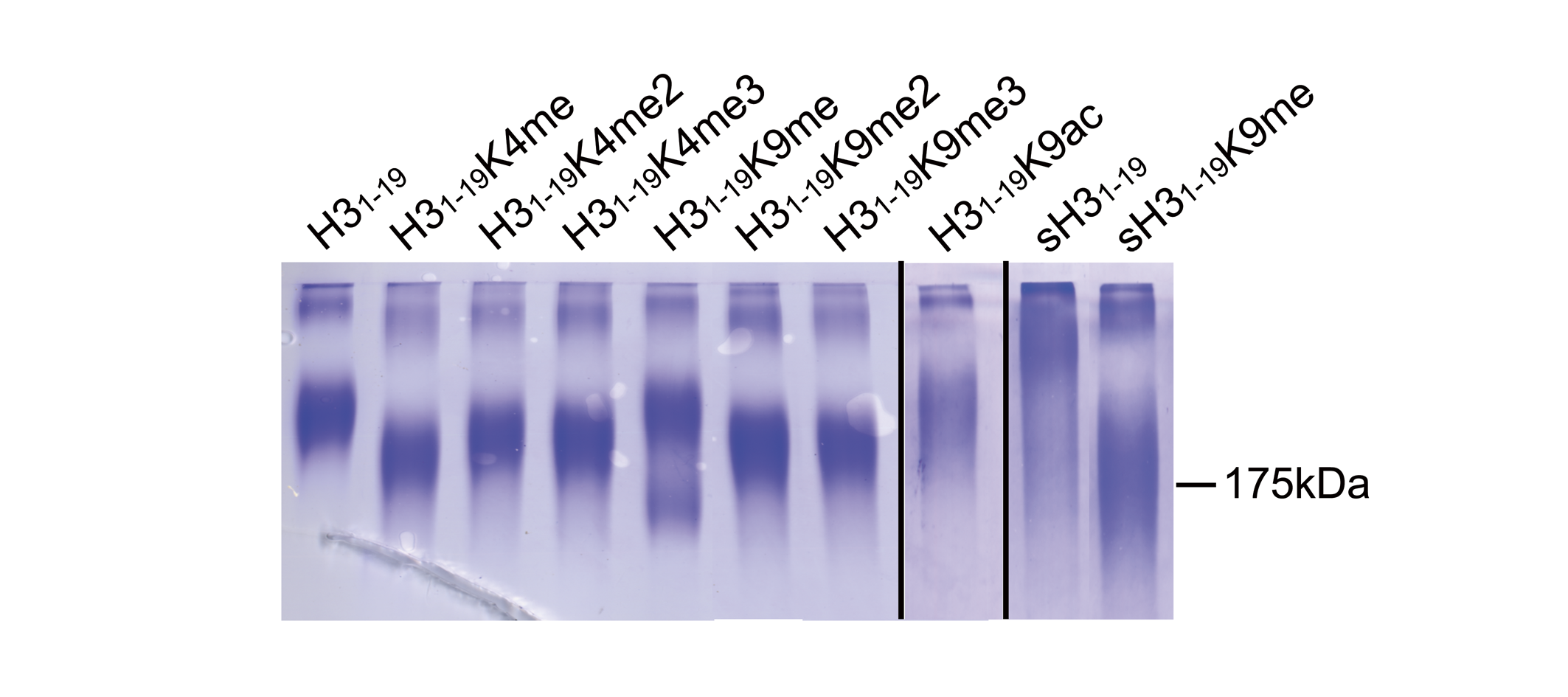

Supplement: Figure S1 — SDS-PAGE of H3 peptides cross-linked to BSA. Synthetic peptide was cross-linked onto BSA. 2 µg (according to the BSA concentration) of each peptide/BSA run on 10% SDS-PAGE under reducing conditions. (TIF) [file pone.0068520.s001.tif]

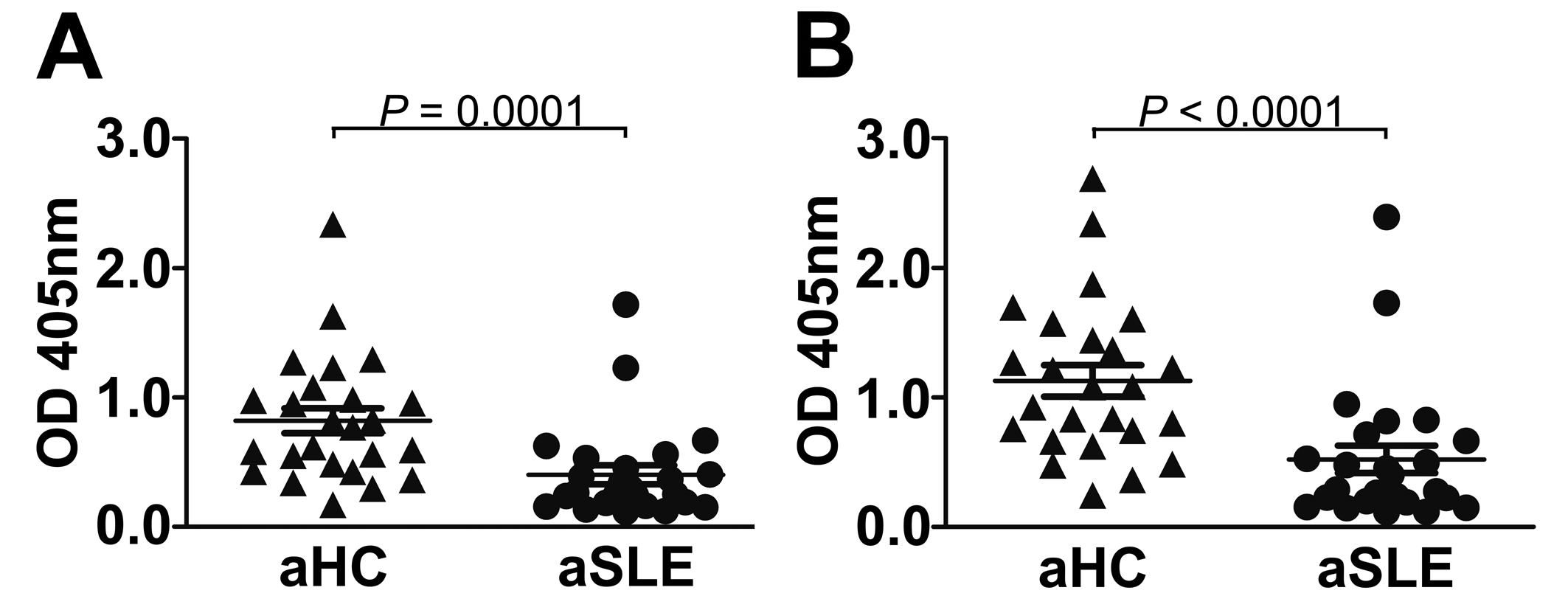

Supplement: Figure S2 — Serum reactivity to H31–19K9me and GGKme. Microtiter plates were coated with H31–19K9me and GGKme conjugated to BSA. Serum samples (n = 25) from the healthy adults or aSLE patients were diluted 1∶100 and tested. KT16 anti-human IgM was used as the primary Ab and HRP-conjugated goat anti-mouse IgG was used as the secondary Ab. Data are expressed as mean±SEM. (A) H31–19K9me coated. (B) GGKme coated. The results are shown as representative of two separate experiments. (TIF) [file pone.0068520.s002.tif]
