# Supplementary material for: Systemic Lupus Erythematosus Patients Contain Significantly Less IgM against Mono-Methylated Lysine than Healthy Subjects
Source: PLoS One. 2013 Jul 16;8(7):e68520. doi: 10.1371/journal.pone.0068520 (PMC3713014; doi:10.1371/journal.pone.0068520)
Supplement: Table S1 — Information about the SLE patients and healthy controls. a, M, male; F, female. Ratios were compared by Chi-square test. b, Ages were compared by Mann-Whitney test. P<0.05 was considered significant. (DOCX) [file pone.0068520.s003.docx]

**Table S1.** **Information about the SLE patients and healthy controls.**

|  | **Pediatric samples** | | |  | **Adult samples** | | |
| --- | --- | --- | --- | --- | --- | --- | --- |
|  | **pHC** | **pSLE** | ***P* value**  **(χ^2^)** |  | **aHC** | **aSLE** | ***P* value**  **(χ^2^)** |
|  |  |  |  |  |  |  |  |
| No. | 62 | 62 |  |  | 75 | 75 |  |
|  |  |  |  |  |  |  |  |
| M:F^a^ | 12:50 | 11:51 | 0.902  (0.050) |  | 11:64 | 11:64 | 1.000  (0.0) |
|  |  |  |  |  |  |  |  |
| Age^b^  (range) | 11.00±3.00  (2 to 14) | 10.69±3.12 (2 to 14) | 0.569 |  | 31.59±9.50  (18 to 53) | 35.00±11.19 (19 to 67) | 0.225 |

a, M, male; F, female. Ratios were compared by Chi-square test.

b, Ages were compared by Mann-Whitney test.

*P* < 0.05 was considered significant.
