# Supplementary material for: Systemic Lupus Erythematosus Patients Contain Significantly Less IgM against Mono-Methylated Lysine than Healthy Subjects
Source: PLoS One. 2013 Jul 16;8(7):e68520. doi: 10.1371/journal.pone.0068520 (PMC3713014; doi:10.1371/journal.pone.0068520)
Supplement: Table S2 — Treatments received by SLE patients at blood collection. GC, Glucocorticoids; IS, Immunosuppressants; AM, Antimalarials; mAbs, monoclonal antibodies; IVIG, Intravenous immunoglobin. a, The number of patients treated with/without that medicine. (DOCX) [file pone.0068520.s004.docx]

**Table S2. Treatments received by SLE patients at blood collection.**

| **pSLE** | | | |  | **aSLE** | | | | |
| --- | --- | --- | --- | --- | --- | --- | --- | --- | --- |
| **ID** | **GC** | **IS** | **IVIG** |  | **ID** | **GC** | **IS** | **AM** | **mAbs** |
|  |  |  |  |  |  |  |  |  |  |
| 1 | + | + | + |  | 1 | + | + | + | + |
| 2 | + | - | - |  | 4 | + | + | + | - |
| 3 | + | + | - |  | 5 | + | + | - | - |
| 4 | + | - | + |  | 6 | + | + | - | - |
| 5 | + | - | - |  | 8 | - | ­- | - | + |
| 7 | + | + | - |  | 11 | + | + | - | - |
| 9 | + | - | + |  | 12 | + | + | - | - |
| 12 | + | + | - |  | 15 | + | + | - | - |
| 13 | + | + | - |  | 18 | + | + | - | + |
| 14 | + | + | - |  | 20 | + | + | - | - |
| 18 | + | + | + |  | 21 | + | - | - | + |
| 30 | + | + | + |  | 22 | + | - | - | - |
| 31 | + | + | - |  | 24 | + | + | - | - |
| 34 | + | - | - |  | 35 | + | + | - | - |
| 41 | + | + | + |  | 38 | + | - | - | - |
| 44 | + | + | + |  | 44 | + | - | - | - |
| 45 | + | - | + |  | 46 | + | + | + | - |
| 46 | + | - | - |  | 48 | + | + | - | - |
| 52 | + | + | - |  | 52 | + | - | - | + |
| 55 | + | + | - |  | 53 | + | + | - | - |
| 56 | + | + | - |  | 54 | - | - | - | + |
| 57 | + | + | - |  | 73 | + | + | + | - |
| 61 | + | + | - |  | 74 | - | - | + | - |
| 62 | + | - | - |  | 98 | + | + | + | - |
| 63 | + | + | - |  | 102 | + | + | - | - |
| 64 | + | + | - |  | 108 | + | + | + | - |
| 92 | + | - | + |  | 112 | + | + | - | - |
| 94 | + | + | + |  | 114 | + | + | - | - |
| 97 | + | + | + |  | 115 | + | + | - | - |
| 100 | + | + | - |  | 129 | + | + | - | - |
| 103 | + | + | + |  | 130 | + | + | - | - |
| 113 | + | + | + |  | 134 | + | + | - | - |
| Summary | 31/0^a^ | 23/9 | 13/18 |  | 136 | + | + | - | - |
|  |  |  |  |  | 141 | + | + | + | - |
|  |  |  |  |  | 146 | + | + | + | - |
|  |  |  |  |  | 149 | + | - | + | - |
|  |  |  |  |  | 153 | + | + | - | - |
|  |  |  |  |  | 154 | + | - | - | - |
|  |  |  |  |  | 155 | + | + | - | - |
|  |  |  |  |  | Summary | 36/3 | 29/10 | 10/29 | 6/33 |

GC, Glucocorticoids; IS, Immunosuppressants; AM, Antimalarials; mAbs, monoclonal antibodies; IVIG, Intravenous immunoglobin.

a, The number of patients treated with/without that medicine.
