# Supplementary material for: Systemic Lupus Erythematosus Patients Contain Significantly Less IgM against Mono-Methylated Lysine than Healthy Subjects
Source: PLoS One. 2013 Jul 16;8(7):e68520. doi: 10.1371/journal.pone.0068520 (PMC3713014; doi:10.1371/journal.pone.0068520)
Supplement: Table S3 — Peptides and their modifications. (DOCX) [file pone.0068520.s005.docx]

**Table S3.** **Peptides and their modifications.**

| **Abbreviations** |  | **Sequences and Modifications** |
| --- | --- | --- |
| H3_1-19_ |  | ARTKQTARKSTGGKAPRKQC |
| H3_1-19_K4me |  | ARTK(me)QTARKSTGGKAPRKQC |
| H3_1-19_K4me2 |  | ARTK(me_2_)QTARKSTGGKAPRKQC |
| H3_1-19_K4me3 |  | ARTK(me_3_)QTARKSTGGKAPRKQC |
| H3_1-19_K9me |  | ARTKQTARK(me)STGGKAPRKQC |
| H3_1-19_K9me2 |  | ARTKQTARK(me_2_)STGGKAPRKQC |
| H3_1-19_K9me3 |  | ARTKQTARK(me_3_)STGGKAPRKQC |
| H3_1-19_K9ac |  | ARTKQTARK(ac)STGGKAPRKQC |
| sH3_1-19_ |  | KTRARAQTKTGSAGKQKPRC |
| sH3_1-19_K9me |  | KTRARAQTK(me)TGSAGKQKPRC |
| GGKme |  | GGK(me)GGSGGSGGSGC |
